# AI-enabled Living Labs: Accelerating innovation in multiple sclerosis care and research

**DOI:** 10.1177/13524585261424136

**Published:** 2026-03-13

**Authors:** Hernan Inojosa, Rebecca Mathias, Anja Dillenseger, Isabel Voigt, Katrin Trentzsch, Julia Steinigen-Fuchs, Katrin Piehler, Stephen Gilbert, Tjalf Ziemssen

**Affiliations:** Center of Clinical Neuroscience, Department of Neurology, Faculty of Medicine and University Hospital Carl Gustav Carus Dresden, TUD Dresden University of Technology, Dresden, Germany; Else Kröner Fresenius Center for Digital Health, TUD Dresden University of Technology, Dresden, Germany; Center of Clinical Neuroscience, Department of Neurology, Faculty of Medicine and University Hospital Carl Gustav Carus Dresden, TUD Dresden University of Technology, Dresden, Germany; Center of Clinical Neuroscience, Department of Neurology, Faculty of Medicine and University Hospital Carl Gustav Carus Dresden, TUD Dresden University of Technology, Dresden, Germany; Center of Clinical Neuroscience, Department of Neurology, Faculty of Medicine and University Hospital Carl Gustav Carus Dresden, TUD Dresden University of Technology, Dresden, Germany; Member of the Ethics Committee, TUD Dresden University of Technology, Dresden, Germany; Quality and Medical Risk Management, Data Security, University Hospital Carl Gustav Carus Dresden, Technical University of Dresden, Dresden, Germany; Else Kröner Fresenius Center for Digital Health, TUD Dresden University of Technology, Dresden, Germany; Center of Clinical Neuroscience, Department of Neurology, Faculty of Medicine and University Hospital Carl Gustav Carus Dresden, TUD Dresden University of Technology, Dresden, Germany

**Keywords:** Living Lab, Learning Health System, multiple sclerosis, artificial intelligence, digital health

## Abstract

The rapid rise of artificial intelligence (AI) and digital health technologies presents new opportunities for personalized care in multiple sclerosis (MS). However, implementation in routine practice is limited by regulatory hurdles, fragmented infrastructure and a lack of agile real-world evaluation methods. Living Labs (LLs) emerge as dynamic environments for advancing MS care and research, supporting early testing and iterative development of digital tools, while fostering structured collaboration among patients, clinicians, researchers and regulators. In this review, we conceptually frame LLs in MS and provide a concrete, clinic-ready implementation framework for AI-enabled application in real-world practice. Using a digital-based voice task as an exemplar with automated feature extraction, we detail integration patterns and define key performance indicators for feasibility, data quality, usability and clinical utility. We show how this co-designed model can generate decision-relevant evidence, may help shorten time-to-action and embed innovation seamlessly into clinical workflows. Finally, we align LL operations with ethical and regulatory standards and outline strategies to responsibly scale across centres.

## Introduction

Digital transformation is reshaping neuroimmunology, with multiple sclerosis (MS) serving as a model disease for testing and implementing novel technologies.^1–4^ Advances in digital biomarkers, patient-reported outcomes (PROs) and artificial intelligence (AI)-driven analytics are gradually being integrated into MS research and slowly into clinical care.^5–7^ However, many innovations remain restricted to pilot projects, often due to regulatory challenges, insufficient validation pathways and a lack of coordination among key stakeholders.^8^

To bridge this gap, Living Labs (LLs) have emerged as clinic-embedded, co-designed settings for participatory innovation.^8–10^ LLs function as protected testbeds for early-stage development and as collaborative ecosystems where people with multiple sclerosis (pwMS), clinicians, developers and regulators co-create solutions. For example, a Dutch LL has successfully used this model to improve eHealth integration in primary care.^10^

While brain health LLs have begun to integrate clinical care and research in broader neurology, their potential within MS-specific settings such as dedicated ‘MS Units’ remains underutilized.^11^ Innovative digital tools demonstrate how human-centred design can create accessible, actionable precision medicine platforms for longitudinal monitoring.^12^ However, to move such innovations from standalone platforms, a structured operational framework is required. LLs offer unique environments to embed high-resolution monitoring tools directly into clinical workflows, while extending data collection and feedback loops into patients’ daily lives.^8^

LLs integrate naturally with Learning Health Systems (LHSs) loops: where cycles of data capture, quality checks and outcome review inform adjustments.^13,14^ Data are written back to electronic health records (EHRs) using auditable, compliant standards of operations (SOPs).^8,15,16^ Together, LLs and LHSs offer a compliant, inclusive approach to accelerate digital innovation in MS.

In this review, we outline key principles of LLs for MS care, illustrate their practical implementation using a visit-integrated speech module as an exemplar and demonstrate their alignment with regulatory compliance requirements.

## Principles of LLs in MS care

**Figure 1.**
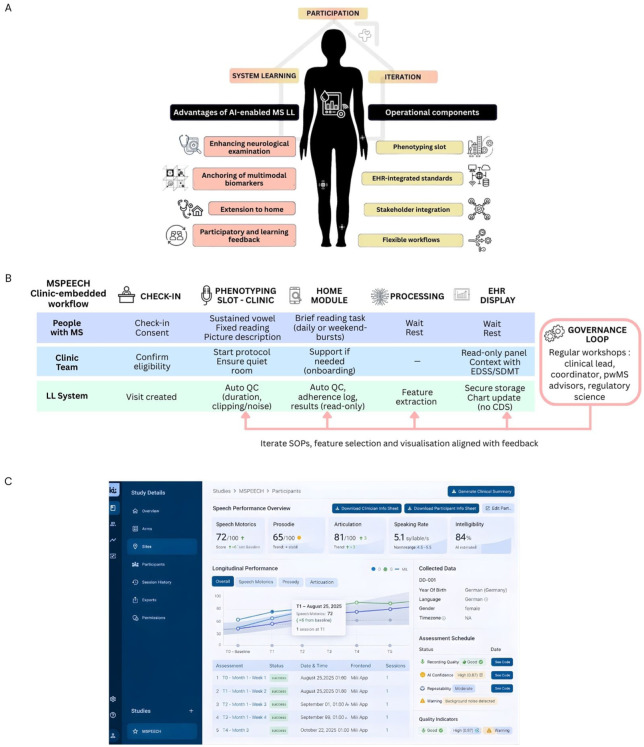
(a) Core principles and operational components of an artificial intelligence (AI)-enabled Multiple Sclerosis (MS) Living Lab (LL): stakeholder participation drives system learning and iteration. Advantages (enhanced neurological examination, multimodal anchoring with imaging/blood biomarkers, extension to home, participatory feedback) link to operational components (phenotyping slot, electronic health record (EHR)–integrated standards in read-only/quality improvement mode, stakeholder integration, flexible workflows). (b) Clinic-embedded speech module workflow: after check-in/consent, a brief in-clinic phenotyping slot (sustained vowel, fixed reading, picture description) is performed; a home module collects short reading tasks. Automatic quality checks (QC) and feature extraction run inside the hospital; results are stored securely and shown the same day in a read-only EHR panel alongside routine measures, with no clinical decision support (CDS). A governance loop – regular workshops with the clinical lead, coordinator, people with MS (pwMS) advisors and privacy/ethics – feeds back into standard operating procedures (SOPs), feature selection and visualization for controlled iteration. (c) Example read-only EHR display/dashboard for the speech module summarizing derived speech metrics, longitudinal trajectories and recording-quality indicators.

Current MS assessment, frequently reliant on ordinal scales, may miss subtle changes. While LLs can be applied broadly to administrative workflows or educational programmes, this review focuses specifically on digitally enabled LLs that use high-resolution technologies to run iterative learning cycles without disrupting care. This section outlines key principles for operationalising LLs in MS (Figure 1(a)).

### Integrating continuous assessment into the neurological examination

Conventional MS disability assessment is limited by subjective evaluations with high inter-rater variability.^17,18^ While short, self-administered digital tasks – such as a brief 3- to 4-minute voice task (see Section 4, Figure 1(b) ) – can offer granular data, their path to sustainable clinical integration is often blocked by a lack of sustainable infrastructure.^19^ Even validated in-clinic tools that correlate well with disability scores present integration challenges.^20^ An LL provides the necessary framework to embed these tools directly within the clinical workflow, bridging the gap from validation to routine use.

By designing a dedicated data pipeline, LLs move standalone assessments into the routine of the neurological exam, ensuring that measurements undergo automatic processing and quality checks for artefact thresholds. Derived features such as speech rate or prosody must be stored traceably with version control to ensure data integrity. This device-agnostic pattern lets centres swap or update tools without re-engineering clinic processes.

Beyond the clinic, the LL framework extends the ‘lab’ to the patient’s daily life to capture real-world data that reflect the disease state between infrequent, episodic office visits. The relevance of this extension is supported by evidence demonstrating that continuous at-home measurements, such as remote monitoring of step counts, can detect disease worsening that traditional care may overlook.^21,22^ To enable this transition, centres can implement a brief onboarding process and short daily tasks supported by automatic quality prompts. Improving adherence in these uncontrolled environments requires a proactive approach, including simple weekly reminders and a monthly review of data-loss patterns. Accepted home data are then linked to the clinical context, while failed captures are flagged for audit to maintain the same rigorous standards applied to in-clinic phenotyping.

During the pilot phase of this integrated model, results can be displayed in a read-only mode indicating they are for research and not yet for diagnosis or treatment (Figure 1(c)). This allows clinicians to view longitudinal trends from both home and clinic alongside routine measures. While any clinical actions continue to follow usual care and clinician judgement, the higher frequency and granularity of these combined measurements facilitate exploratory analyses.

### Multimodal anchoring with imaging and blood biomarkers

Digital innovations must be interpreted against established references like magnetic resonance imaging (MRI) or biofluid markers to avoid overclaiming and anchor functional outcomes (e.g. from wearables) to underlying pathobiology.^23^ In the LL framework, multimodal AI approaches may support care-relevant tasks detecting patterns or latent signals, predicting progression or supporting earlier intervention. However, robust anchoring requires strict operational definitions to ensure data from different timescales, such as continuous sensor streams versus annual MRI scans, can be reliably integrated. To achieve this, the LL must enforce predefined validity rules such as temporal alignment or ‘acceptable missingness’ thresholds.

This approach is exemplified by the NeuroPredict platform, which employs a timestamp alignment and multimodal fusion across heterogeneous streams, or the Open MS BioScreen, which validated digital features alongside ‘hard’ anchors within a unified dashboard.^12,22^

### Participatory learning and feedback

The hallmark of a ‘Living’ Lab is its cyclical, participatory design for continuous learning (Figure 1(a)). Unlike static research protocols, LLs are dynamic ecosystems evolving via stakeholder feedback. This co-creation ensures innovations are practical and aligned with real-world needs. Capturing user perspectives early increases the clinical value of digital health solutions. We illustrate this cycle using a visit-integrated speech module MSPEECH in Section 4 (Figure 1(b)).

Each cycle prespecifies capture (what/when/how), quality rules and re-take policy, clinician display and allowed actions (note comment, test order, referral), outcome review (feasibility, adherence, data quality, usability, and time-to-action) and concrete changes to SOPs or user interface for the next release. By recording minutes and version changes, iterations are kept explicit, auditable and safe. The MS LL thus operates as a learning system–accumulating data and experience to improve its outputs. This philosophy of continuous improvement and stakeholder co-involvement makes a LL ‘Living’.

## Implementing LLs and LHS in real world

### Co-design, governance and roles

Central to the LL model is co-design, wherein stakeholders collaborate from the earliest stages of tool development.^9^ In practice, this means involving pwMS in defining measurement priorities, usability testing and refinement cycles.^24^ This co-design process is critical for success in chronic diseases like MS. For instance, the ‘iManage’ app for adolescents with sickle cell disease serves as example of LL co-design, demonstrating how patient involvement leads to high acceptability and tailored use.^25^ In neurology, highly complex, multimodal ecosystems seek to combine patient-reported data with imaging, genomics and passive sensor streams.^12,22^ Applying the participatory principles to these sophisticated technical architectures is essential to ensure they remain sustainable and grounded in real-world patient needs.

Each MS LL runs on named roles, short cadences and versioned SOPs. This may include:

Clinical lead (neurology): owns clinical appropriateness and escalation paths; signs SOPs.
LL coordinator (operations): schedules the phenotyping slot, tracks KPIs, runs the weekly stand-up, maintains SOPs.
Data/IT liaison: implements capture, performance indicators, EHR write-back, access control, logging.
Privacy/ethics officer: consent language, retention, audit readiness.
PwMS advisors: compensated; review burden/usability and vote on changes.

We suggest a 20-minute weekly stand-up (coordinator, engineer, clinic rep) and a 60-minute quarterly board meeting (adding clinical lead, privacy officer and pwMS advisors) to approve material changes. To maintain this participatory workflow, LLs should balance data privacy, transparency and ethical oversight (see below). Informed consent processes emphasize how real-world data will be used, and participants retain the ability to view or withdraw their data at any point.^8^ Regular user-feedback loops and advisory boards as dynamic feedback structure distinguish LLs from traditional pilot studies or registries, which often lack real-time adaptability.

### Visit workflow (phenotyping slot)

The in-clinic ‘phenotyping slot’ is a fixed 5- to 10-minute block embedded in the routine visit. At check-in, eligibility and consent are confirmed in the visit template (Figure 1(b)). The patient is guided through short, scripted tasks using clinic-approved devices in a dedicated room. Staff involvement is limited to starting the protocol and confirming completion. During the pilot phase, the panel is limited to a research/quality-improvement view: values are displayed for context only, and all diagnostic and treatment decisions follow usual care pathways without automated recommendations or thresholds. Operational targets should remain pragmatic (approximately 1 minute setup, 6 minutes capture, 2 minutes feedback) and be monitored as workflow metrics rather than performance claims.

Each innovation module (e.g. voice, gait, dexterity) should have a clear version covering capture, preprocessing/features and any associated model. Before display, it should be checked offline for repeatability and face validity and documented in a one-page model note. The first deployment should remain read-only/QI (not for diagnostic or treatment use). Monthly, simple metrics should be reviewed and two consecutive misses should trigger a fix or temporary revert. The last stable version should be retained for rapid rollback. Moving to clinical use requires local approval and compliance with applicable medical device rules.

### Interoperability and data integration

All captures should be handled inside the hospital environment and written back in a structured, EHR-compatible format so clinicians can view results within the chart. Raw sensor files (e.g. audio) are to be stored securely; the chart receives concise feature summaries linked to the visit and the capturing device, with version and operator recorded for traceability. To ensure structured, EHR-compatible data write-back and interoperability, LLs should leverage industry-standard application programming interfaces and protocols, such as Fast Healthcare Interoperability Resources (FHIR) for exchanging clinical and administrative data.^26^

For a visit-integrated voice-module, raw audio is retained in secure storage; derived features (e.g. speech rate, articulation rate, pause metrics, prosody) are written back as structured entries linked to the visit and capture device, with source and version information recorded for traceability (see Section 4 for the exemplar).

### Regulatory and ethical framework

Because flexibility is the defining feature of an LL, a clear regulatory and ethical frame is essential to make iterations safe, auditable and scalable. The inherent LL flexibility presents challenges when interfacing with the stringent requirements of regulatory frameworks, particularly those governing medical devices, digital health tools and data privacy in Europe and beyond. Regulatory bodies, guided by instruments such as the EU Medical Device Regulation (MDR), General Data Protection Regulation (GDPR), US Food and Drug Administration (FDA) and the EU AI Act, prioritize transparency, scientific validity and traceability.^27–29^ These are complemented by foundational ethical documents, mandating strict oversight of any intervention involving human participants. LLs, by contrast, often rely on iterative, adaptive methodologies difficult to reconcile with rigid regulatory pathways.^30–32^

Bridging this gap demands carefully designed and complementary strategies. First, MS LLs can join flexibility with compliance by implementing digital monitoring infrastructures that support traceability, reproducibility and safety auditing.^8^ These systems can generate high-quality data streams that align with regulatory documentation standards, such as technical files or post-market surveillance requirements under the MDR. Their built-in feedback loops also facilitate timely protocol adjustments, while preserving transparency for both clinicians and oversight bodies.

Ethical compliance is central. Rather than bypassing ethical review, LLs in MS should operationalize dynamic consent mechanisms, quality control and patient communication. These measures ensure participant protection while maintaining the adaptability needed to improve tools iteratively. Because many MS LL evaluations are embedded in routine care and pose minimal risk, institutional review boards may classify them as quality improvement (QI) rather than formal research.^14^ Under this designation, a nuanced distinction is required: certain pragmatic approaches – such as phased implementation of digital tools when full deployment is not feasible – have been discussed within QI frameworks to balance adaptability with accountability in care settings .^14^ In contrast, randomization remains ethically controversial in these contexts and is often avoided due to concerns around informed consent and oversight.

The concept of Technology Readiness Levels (TRLs) provides a useful framework to align innovation with regulatory stringency.^33^ In early TRLs (1–2), LLs can focus on exploratory interaction (qualitative feedback on usability, feasibility and engagement) typically with minimal regulatory burden. As projects mature into TRLs 3–9, regulatory expectations increase sharply. MS LLs thus fulfil a regulatory-relevant dual function: they offer a protected, quality-controlled environment for iterative development and feasibility testing within clinical settings. Here, LLs can propose evaluation envelopes (predefined ranges within which adaptation is permitted) to preserve iterative development while remaining within ethical and regulatory bounds. In the European context, in-house device development within health institutions is permitted, provided the devices meet specific pwMS needs not addressed by commercial tools and adhere to internal quality standards.^8,27^ This provision is particularly relevant for MS Units developing digital modules, when no certified alternatives exist. It allows innovations to be embedded within routine care while maintaining safety and traceability.

An additional mechanism is the use of regulatory sandboxes, in which developers and regulators engage in real-time dialogue and jointly define the scope of flexible, supervised experimentation.^8^ This model can be instrumental for MS-focused innovations such as AI-driven symptom tracking or personalized prediction models, where conventional evaluation frameworks may not yet exist. By combining the adaptivity of LLs with the structured accountability of regulatory sandboxes, MS units can test and refine technologies under realistic conditions.

In summary, MS LLs can operate within, not outside, regulatory and ethical frameworks by embedding continuous monitoring, traceable development processes and dynamic stakeholder engagement. This approach ensures that promising digital tools, many of which begin in academic or clinical settings, can ultimately reach certification and scale across broader MS care ecosystems.

## Speech-based exemplar: MSPEECH

MSPEECH is a clinic-embedded module within the Dresden MS LL being piloted as a brief speech module with an optional at-home extension for low-burden monitoring, using automated feature extraction from structured voice tasks (Figure 1(b)). After check-in, participants complete a 3- to 4-minute, on-screen–prompted protocol in a quiet room (sustained vowel, fixed reading passage, picture description). Basic quality controls (minimum duration, clipping/noise) run automatically; if a check fails, a re-take may be performed according to local standard operating procedures. Acoustic–prosodic features (e.g. speech rate, articulation rate, pause metrics, prosody) are computed, and summarized results are available in a read-only view for situational awareness (Figure 1(c)); any therapy decisions continue to follow usual care and clinician judgement, and the module is not certified for clinical decision support.

The system is being designed, implemented and evaluated across three co-creative cycles. As of the current cut-off, 25 pwMS have been recruited for MSPEECH. In parallel, participants use wearables and app-based tasks at home to continue data collection under real-world conditions. Workshops with pwMS and study staff review burden, instructions and visualization and approve any material changes.

Early qualitative feedback indicates that the app generally works as intended; isolated issues were reported where follow-up test modules did not unlock or reminders did not trigger. These items are being tracked for root cause analysis and will inform the next iteration of SOPs, feature selection and panel visualization within the governance loop (Figure 1(b)). A secure back-end platform aggregates these streams and generates visual dashboards accessible to clinicians and pwMS alike. The platform also supports protocol-specific modules for evaluating new tools under regulatory frameworks such as the EU MDR and EU AI Act.^8^ To date, the Dresden MS LL has supported selected co-designed projects, including acoustic biomarkers of fatigue or using federated learning approaches.^34,35^ A key next step is to report empirical feasibility, data quality and usability outcomes from MSPEECH and related modules.

## Future directions and conclusion

LLs offer a powerful framework to bridge MS care, research and digital innovation. Critically, the innovation lies not only in the digital tools, but also in the operational platforms themselves: resilient integrated ecosystems that embed these tools into routine practice. By enabling real-world co-creation, continuous learning and regulatory-aligned validation, LLs can accelerate biomarker discovery and personalize disease monitoring. A key step for scaling the LL model is to build multi-centre consortia. This involves adopting lessons from successful large-scale LHSs, federated digital biomarker platforms or LLs in other fields.^9,36^ As participation and data integration evolve, LLs may help shape a more adaptive, patient-centred model for MS care.
